# Adequacy of Antenatal Care during the COVID-19 Pandemic: Observational Study with Postpartum Women

**DOI:** 10.1055/s-0041-1741450

**Published:** 2022-02-17

**Authors:** Margot Marie Martin, Roxana Knobel, Vitor Nandi, Jessica Goedert Pereira, Alberto Trapani Junior, Carla Betina Andreucci

**Affiliations:** 1 Divisão de Saúde da Mulher, Hospital Universitário Polydoro Ernani de São Thiago, Florianópolis, SC, Brazil; 2 Departamento de ginecologia e obstetrícia, Universidade Federal de Santa Catarina, Florianópolis, SC, Brazil; 3 Programa de residência médica em ginecologia e obstetrícia, Hospital Regional Homero de Miranda Gomes, São José, SC, Brazil; 4 Departamento de medicina, Universidade Federal de São Carlos, São Carlos, SP, Brazil

**Keywords:** prenatal care, primary health care, access to health services, Covid-19, cuidado pré-natal, atenção primária à saúde, acesso aos serviços de saúde, Covid-19

## Abstract

**Objective** The present study aimed to evaluate the antenatal care adequacy for women who gave birth at the University Hospital of Santa Catarina in Florianopolis (Brazil) during the COVID-19 pandemic, and to evaluate the association of adequacy with sociodemographic, clinical, and access characteristics.

**Methods** Data were collected between October and December 2020, including 254 patients who delivered in the University Hospital from Federal University of Santa Catarina and answered our questionnaires. Additional data were obtained from patients' antenatal booklets. Antenatal care was classified as adequate, intermediate, or inadequate according to the number of appointments, gestational age at the beginning of follow-up, and tests results. We carried out a descriptive statistical analysis and a bivariate/with odds ratio analysis on maternal sociodemographic, clinical and health access variables that were compared with antenatal adequacy.

**Results** Antenatal care was considered adequate in 35.8% of cases, intermediate in 46.8%, and inadequate in 17.4%. The following maternal variables were associated with inadequate prenatal care (intermediate or inadequate prenatal care): having black or brown skin colour, having two or more children, being of foreign nationality, not being fluent in Portuguese, and using illicit drugs during pregnancy; the clinical variables were more than 6 weeks between appointments, and not attending high-risk antenatal care; as for access, the variables were difficulties in attending or scheduling appointments, and attending virtual appointments only.

**Conclusion** In a sample of pregnant women from a teaching hospital in Florianópolis during the COVID-19 pandemic, antenatal care was considered adequate in 35.8%, intermediate in 46.8%, and inadequate in 17.4% of cases.

## Resumo

**Objetivo** O objetivo deste estudo foi avaliar a adequabilidade do pré-natal de puérperas atendidas no hospital universitário da Universidade Federal de Santa Catarina, em Florianópolis, durante a pandemia de COVID-19 e avaliar a associação de características sociodemográficas, clínicas e de acesso com essa adequabilidade.

**Métodos** Este estudo foi realizado de outubro a dezembro de 2020, com 254 puérperas que tiveram seus partos no hospital universitário. Os dados foram obtidos a partir de questionários respondidos pelas pacientes e dos seus cartões de pré-natal e prontuários para obter demais dados clínicos. O pré-natal foi classificado como adequado, intermediário ou inadequado segundo o número de consultas, idade gestacional ao início do pré-natal, e realização de exames. Inicialmente, se realizou uma análise estatística descritiva e, após, bivariada/com razão de chance quanto às variáveis maternas sociodemográficas, clínicas, e de acesso a saúde comparados com adequabilidade do pré-natal.

**Resultados** O pré-natal foi considerado adequado em 35,8%, intermediário em 46,8% e inadequado em 17,4% dos casos. Estiveram associados a uma assistência pré-natal não-adequada (pré-natal intermediário ou inadequado) as seguintes variáveis maternas: cor de pele preta, parda, ou indígena, ter dois ou mais filhos, ser de nacionalidade estrangeira, não possuir fluência em português, uso de drogas ilícitas durante a gestação; as variáveis clinicas foram: lacuna de mais de 6 semanas entre consultas e não ser atendida em pré-natal de alto risco; quanto a acesso, as variáveis foram: dificuldade de ir e de agendar as consultas e ter tido consultas virtuais.

**Conclusão** Em uma amostra de gestantes de um hospital universitário de Florianópolis durante a pandemia do Covid-19, a assistência pré-natal foi considerada adequada em 35,8%, intermediária em 46,8%, e inadequada em 17,4% dos casos.

## Introduction

Adequate antenatal care (ANC) can reduce complications during childbirth and in the postpartum period, contributing to a decrease in maternal and infant mortality/morbidity. 1 2 3 About 4,000 infant and neonatal deaths could have been prevented by proper antenatal care during 2014 in Brazil, corresponding to 40% of all deaths. 3 Several affections that still have high rates in the country, such as preterm birth and HIV or syphilis vertical transmission, are related with inadequacy of antenatal care. 4 5

In the last few decades, several low- and high-risk pregnancies antenatal care protocols were proposed in Brazil. The guidelines were designed to instruct best practices, establishing the scope of primary care health professionals, therefore ensuring adequate obstetric care. Early beginning of antenatal follow-up during the first trimester of pregnancy, attendance to at least six antenatal appointments, and basic laboratory tests are among the minimum recommendations. 2 3 4 5

Sociodemographic differences and local context should be considered for assessing and planning health policies. Antenatal care coverage is extensive in overall Brazil, with high adhesion within all country regions. 4 However, care adequacy is low, depending on pregnant women's characteristics. 2 3 6 Before the emergence of the COVID-19 pandemic, the proportion of adequate antenatal care among all Brazilian pregnant women in 2012 (at least 6 appointments) was only of 73%. 3 Additionally, only three quarters of women had early beginning of antenatal care follow-up, and care adequacy was lower for younger women, and for black women from the North and Northeast regions. 5 Pregnancy in adolescents, poverty, low literacy, parity, Brazilian region origin, living in municipalities with low HDI, and not being white are cited as possible barriers to access antenatal care. 2 3 4 6

The 2020 worldwide COVID-19 pandemic caused health systems overload, as well as transportation and free movement restrictions, leading to anxiety amidst the population. 7 As soon as the pandemic hit Brazil, routine antenatal consultations were suspended, although qualified prenatal access is considered essential in health emergencies. 8 Official recommendations were vague and paradoxical, including to avoid face-to-face appointments for low-risk pregnancy, although maintaining the attendance for high-risk cases. 9 There was restriction for outpatient care during the pandemic, negatively impacting the health of pregnant women and their families. 10 Reduction of public transportation, irregular operation of health units, and fear of contagion by women and health professionals also hampered antenatal access, causing delays in seeking and obtaining care. 7 The COVID-19 pandemic affects vulnerable populations more severely, exacerbating inequalities in health access. 11

Quantifying access and adequacy of antenatal care during the pandemic may provide acknowledgement of barriers and risk factors associated with inadequate obstetric care. The results may allow us to recognize which women are at higher risk of inadequate care, as well as to promote inclusive care policies, improving maternal and neonatal outcomes.

The objectives of our study are to evaluate the antenatal adequacy in postpartum women who gave birth at the university hospital of Universidade Federal de Santa Catarina (HU-UFSC) during the COVID-19 pandemic, and to evaluate the association of adequacy with sociodemographic, clinical and health access characteristics.

## Methods

This is a cross-sectional observational study including postpartum women admitted to Hospital Universitário Polydoro Ernani São Thiago at HU-UFSC. The facility is a public referral tertiary hospital within the *Empresa Brasileira de Serviços Hospitalares* (EBSERH) network, in Florianópolis, Santa Catarina, Brazil. The hospital is not a COVID-19 referral center.

Postpartum women who gave birth at the HU-UFSC were interviewed from October 13 to December 30, 2020, during the COVID-19 pandemic. The interview was carried out between 1 and 2 days after birth (for vaginal birth and caesarean section, respectively), just before the patient's discharge.

Women who birthed babies weighting > 500 g and/or with gestational age > 22 weeks were included in the study, recruited from rooming-in infirmary. The exclusion criteria were severe mental illness, home births, and refusal to participate. In case the woman was not in the infirmary or was asleep at the first interview attempt, a second approach followed. If not found or unavailable, the woman was excluded from the sample. Women who did not fulfil the variable of interest, and those for whom less than 50% of the variables were analyzed were also excluded.

Sample size was calculated with population parameter estimation approach, 95% CI, margin of error of 5%, and expected proportion in the population of 65.8%. 5 A sample of 122 women would be necessary for antenatal quality care evaluation. To assess factors associated with adequacy of care in a broader approach, we decided to include all participants who met the inclusion criteria and filled out the variable of interest.

Participants received a self-completion questionnaire specially prepared for the project containing multiple-choice and open-ended questions. The questionnaire was pretested in a similar sample and was available in Portuguese, English, Spanish, and French. The instrument could be filled out with the help of one of the researchers, if requested. Additionally, we collected information from hospital charts and from antenatal booklets. Data on antenatal consultations were obtained through antenatal booklets and information provided by the women only, since there is no means for accessing primary care follow-up through the hospital. The included variables were identification, socioeconomic status, personal morbid history, lifestyle, and information about antenatal care. From the medical chart and antenatal booklet, we obtained information on antenatal follow-up, obstetric data, and previous conditions.

The present study is part of the research “Obstetric and postpartum complications during the COVID-19 epidemic”, No 5543120.7.0000.0121, and it was approved at the UFSC human research ethics committee, according to requirements for studies involving human beings at CNS Resolution 466/12 and their complementary resolutions.

Quality of antenatal care was the dependent variable. We used Kessner index 2 12 to analyze whether antenatal care was adequate, intermediate, or inadequate. 2 4 5 13 Having at least basic laboratory tests (HIV, syphilis, and routine urine) at the third trimester of pregnancy during the pandemic was considered an adequacy criterion, adapted from Silveira et al. 14 Thus, antenatal care was considered adequate if a) the first appointment happened before the 16 ^th^ week of pregnancy, b) the woman attended to more than 6 consultations, and c) laboratory tests for HIV, syphilis, and routine urine were performed at the last trimester of pregnancy. Antenatal care was inadequate if presenting 1 of 2 characteristics: either less than 3 appointments, or late beginning of follow-up (after 27th week of pregnancy). Cases that did not meet adequate or inadequate criteria were considered intermediate. Patients who had no antenatal care follow-up were considered inadequate. The dependent variable “adequacy of antenatal care” was transformed into dichotomous (adequate or inadequate) to calculate associations and odds ratio.

The independent sociodemographic and health variables were maternal age, literacy, skin color, paid work engagement, social class, nationality, fluency in Portuguese, primiparity, living with a partner/with children, substance use during pregnancy (alcohol, smoking, or illicit drugs), social isolation due to pandemic measures, previous comorbidities, and suspected or diagnosed COVID-19.

We considered the following characteristics for antenatal care and health access: number of appointments and gestational age at the beginning of follow-up, more than 6 weeks of interval between appointments, high-risk pregnancy (with referral to high-risk unit-PNAR), hospitalization during pregnancy, private health insurance, antenatal care location, laboratory tests (syphilis, HIV, and routine urine during the last trimester of pregnancy), reported obstacles to schedule or attend to appointments, teleconsultations, and unexpected expenses during antenatal care.

We applied the Brazilian economic classification criterion for determining the participant's socioeconomic class. 15 The participants were classified as class A with average income of U$ 4,251.97; B1, with average income of U$ 1,951.77; B2, with average income of U$ 1,020.01; C1, with average income of U$ 569,46; C2, with average income of 338.01; D/E, with average income of U$ 152.28 (exchange rate calculated in April 2021).

Social isolation during the COVID-19 pandemic was investigated by asking women whether they avoided crowds, worked out of home, routinely left the house, had close contacts with people with whom they do not cohabit and used public transportation. We classified social isolation as complete if the pregnant woman refrained from all aforementioned exposures, as partial when she avoided the majority, and as no social isolation if she was exposed to all situations.

The data were analyzed using the IBM SPSS Statistics for Windows, Version 27.0 (IBM Corp., Armonk, NY, USA). We applied descriptive statistics (absolute and relative frequency, median and standard deviation) for all variables. To compare sociodemographic characteristics and health habits to obtain antenatal care adequacy in three categories (adequate, intermediate, and inadequate), the chi-squared test and the Fisher exact test were applied. Associations and odds ratios between the dependent variable (adequate or inadequate antenatal care), as well as other variables of interest, were analyzed using binary logistic regression, with a 95% confidence interval (95% CI). For the adjusted regression analysis, we used variables that had *p* < 0.250 in the crude analysis only, in addition to possible confounding factors, such as age and social class. We adopted 5% significance level for all analyses.

## Results

**Fig. 1:**
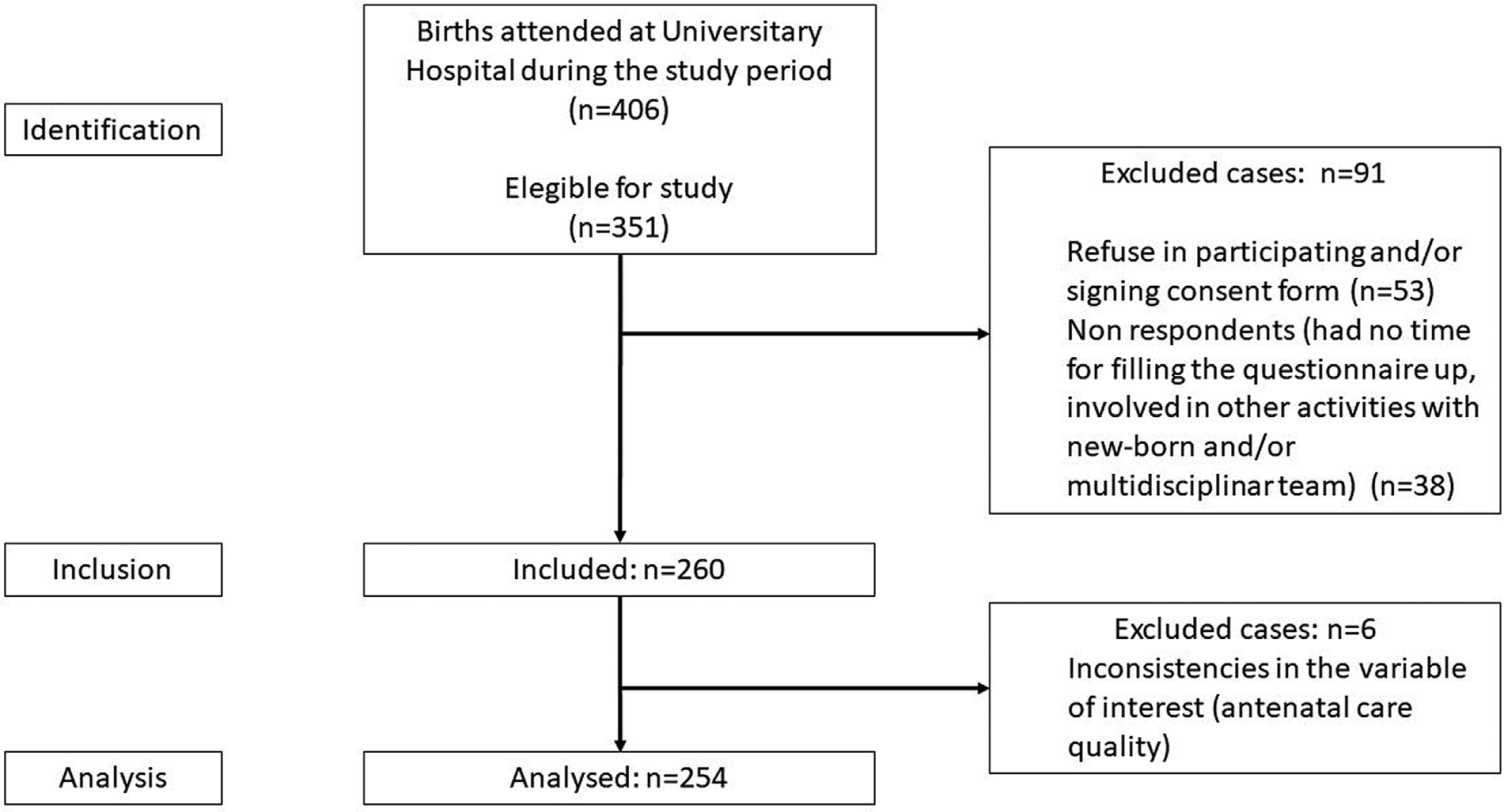
Flowchart of subject's selection.

From October 10 to December 30, 2020, 351 women were deemed eligible for the study. Of these, 91 did not participate: 53 did not wish to answer the questionnaire, and 38 agreed, but did not complete the questionnaire for several reasons (engaged in baby care or in multidisciplinary appointments or did not have time before hospital discharge). Six questionnaires were excluded from the analysis due to inconsistencies in the variable “adequacy of antenatal care”. The final sample consisted of 254 patients ( Figure 1 ).

Table 1 shows the distribution of participants according to sociodemographic characteristics and adequacy of antenatal care. We found 119 women with intermediate antenatal care (46.9% of the sample), 91 (35.8%) with adequate antenatal care (35.8% of the sample), and 44 with inadequate care (17.3% of the sample). Few women did not answer some items in the questionnaire, thus reducing the number of responses to the variables. However, women who did not answer any of the questions were excluded from the analysis, as they did not fulfil the variable of interest. The variables that showed significant differences were skin color, not being primiparous, and foreign nationality.

**Table 1:** Sociodemographic characteristics and health habits of study participants

Variables	Antenatal care follow-up			*P* -value*
	Adequate n (%)	Intermediate n (%)	Inadequate n (%)	
Age (n = 224)				
Younger than 20 years	3 (20.0)	8 (53.3)	4 (26.7)	0.686
20 to 34 years	58 (36.0)	77 (47.8)	26 (16.1)	
35 years or older	17 (35.4)	24 (50.0)	7 (14.6)	
Literacy (n = 222)				
0 to 8 education years	6 (30.0)	11 (55.0)	3 (15.0)	0.529
9 to 12 education years	45 (31.3)	72 (50.0)	27 (18.8)	
13 years or above	25 (43.1)	26 (44.8)	7 (12.1)	
Social class (n = 223)				
B1/B2/C1	49 (40.2)	56 (45.9)	17 (13.9)	0.133
C2/D/E	28 (27.7)	53 (52.5)	20 (19.8)	
Skin color (n = 222)				
White/Asian	60 (42.9)	55 (39.3)	25 (17.9)	0.001
Black/brown	17 (20.7)	53 (64.6)	12 (14.6)	
Primiparous (n = 254)				
Yes	38 (34.9)	59 (54.1)	12 (11.0)	0.037
No	53 (36.6)	60 (41.4)	32 (22.1)	
Paid work (n = 206)				
Formal	35 (38.0)	46 (50.0)	11 (12.0)	0.489
Informal	18 (41.9)	19 (44.2)	6 (14.0)	
No paid work	22 (31.0)	34 (47.9)	15 (21.1)	
Lives with partner (n = 210)				
Yes	67 (38.1)	85 (48.3)	24 (13.6)	0.473
No	10 (29.4)	17 (50.0)	7 (20.6)	

Table 2 shows the distribution of women according to sociodemographic characteristics and health habits, as well as having received adequate or inadequate antenatal care (intermediate and inadequate). Both crude and adjusted analyses showed that being black or brown-skinned were risk factors for inadequate antenatal care. In addition, these women are three times more likely to receive inadequate antenatal care. The variable low social class (C2/D/E) showed a borderline association in the crude analysis, which did not remain in the adjusted analysis. Regarding literacy, most of the sample was of white women who completed high school. The average age was 28 years (in full years), with a standard deviation of 6 years, with most women being in the 20 to 34 age group. No participant was from socioeconomic class A, and only five were from class B1. Thirty-six women were classified as D/E. For the entire sample, the beginning of antenatal follow-up was on average at the 12 ^th^ week of gestation (standard deviation of 7 weeks), and women had an average of 7 appointments (standard deviation of 3 consultations). A total of 63% of women started antenatal care follow-up before the 16 ^th^ week of gestation, 48.4% before the 12 ^th^ week, and 34.3% had 6 or more appointments (not tabulated data).

**Table 2:** Distribution of women according to sociodemographic characteristics and health habits by adequacy of antenatal care

Variables	Antenatal follow-up		Crude OR (CI95%)	Adjusted OR (CI95%)
	Adequate n (%)	Inadequate n (%)		
Age				
Younger than 20 years	3 (20.0)	12 (80.0)	2.25 (0.61-8.31)	
20–34 years	58 (36.0)	103 (64.0)	1	
35 years or older	17 (35.4)	31 (64.6)	1.03 (0.52-2.01)	
Literacy				
0–8 education years	6 (30.0)	14 (70.0)	1.06 (0.38-2.94)	
9–12 education years	45 (31.3)	99 (68.8)	1	
13 years or above	25 (43.1)	33 (56.9)	0.60 (0.32-1.12)	
Skin color				
White/Asiatic	60 (42.9)	80 (57.1)	1	1
Black/brown	17 (20.7)	65 (79.3)	2.87 (1.53-5.39)*	2.99 (1.49-6.00)*
Primiparous				
Yes	38 (34.9)	71 (65.1)	1.08 (0.64-1.81)	
No	53 (36.6)	92 (63.4)	1	
Paid work				
Yes	57 (37.7)	94 (62.3)	1	
No	20 (31.7)	43 (68.3)	1.30 (0.70-2.43)	
Lives with partner				
Yes	67 (38.1)	109 (61.9)	0.68 (0.31-1.51)	
No	10 (29.4)	24 (70.6)	1	
Lives with own children				
Yes	42 (35.3)	77 (64.7)	1.15 (0.65-2.02)	
No	35 (38.5)	56 (61.5)	1	
Foreign				
Yes	2 (18.2)	9 (81.8)	2.60 (0.55-12.31)	2.47 (0.26-23.94)
No	89 (36.6)	154 (63.4)	1	1
Speaks Portuguese				
Yes	89 (36.2)	157 (63.8)	1	
No	2 (25.0)	6 (75.0)	1.70 (0.34-8.61)	
Social class				
B1/B2/C1	49 (40.2)	73 (59.8)	1	1
C2/D/E	28 (27.7)	73 (72.3)	1.75 (0.99-3.08)	1.23 (0.64-2.35)
Previous comorbidities				
Yes	26 (34.7)	49 (65.3)	1.04 (0.58-1.86)	
No	54 (35.5)	98 (64.5)	1	
Alcohol abuse				
Yes	9 (26.5)	25 (73.5)	1.71 (0.76-3.88)	1.89 (0.75-4.75)
No	69 (38.1)	112 (61.9)	1	1
Smoking				
Yes	7 (27.0)	21 (75.0)	1.84 (0.74-4.54)	1.31 (0.49-3.49)
No	71 (38.0)	116 (62.0)	1	1
Illicit drugs				
Yes	5 (33.3)	10 (66.7)	1.15 (0.38-3.49)	
No	73 (36.5)	127 (63.5)	1	

Abbreviation: OR, odds ratio.

* *p* < 0.05; Hosmer-Lemeshow = 0.452.

The variables of antenatal care characteristics are distributed according to adequacy in table 3 . The number of appointments and gestational age at the beginning of antenatal follow-up were used to classify the studied variable (adequacy of antenatal care); therefore, statistically significant differences were expected. The variables were maintained in the regression model, and the result showed that an increase by one appointment decreased in 34% the chance of inadequate antenatal care (0.66; 95% CI: 0.55–0.79). In the same way, each delay of one week in starting antenatal follow-up increased in 1.17 times the chance of inadequate antenatal care (95% CI: 1.06–1.29). In addition, an interval longer than 6 weeks between appointments, not attending high-risk unit, obstacles to schedule or to attend to appointments, and having virtual consultations were risk factors for not receiving adequate antenatal care, while exclusively private health insurance was a protective factor. In the adjusted analysis, only obstacles to schedule and to attend appointments and virtual consultations remained significant (both increasing the chance of inadequate antenatal by three times). Seventy-two women did not have third trimester laboratory tests (HIV, syphilis, and routine urine); thus, 50 of them were classified as intermediate, and 22 as inadequate (data not shown). Out of 254 women, 95 reported unexpected expenses (they paid for appointments, laboratory, and imaging tests), corresponding to 45% of the sample. Among six women who had no antenatal follow-up (included in the inadequate antenatal group), three claimed they were unable to schedule appointments at a primary health facility in Florianópolis, and others claimed as reasons for abandoning antenatal care follow-up unwanted pregnancies, late diagnosis, and moving out of the country (data not shown).

**Table 3:** Antenatal characteristics according to adequacy of offered care

Variables	Antenatal care follow-up		Crude OR (CI95%)	Adjusted OR** (CI95%)
	Adequate median (SD)	Inadequate median (SD)		
Number of appointments	9 (1.99)	6 (2.99)	0.66 (0.58–0.75) ^&^	0.66 (0.55–0.79) ^&^
Gestational age at beginning of follow-up	9 (3.03)	12 (8.56)	1.13 (1.07–1.20) ^&^	1.17 (1.06–1.29)*
	**Adequate** **n (%)**	**Inadequate** **n (%)**		
Over 6 weeks interval between appointments				
Yes	35 (25.9)	100 (74.1)	2.92 (1.68–5.07) ^&^	1.84 (0.66–5.16)
No	49 (50.5)	48 (49.5)	1	1
Referral to high-risk unit				
Yes	32 (43.2)	42 (56.8)	1	
No	59 (32.8)	121 (67.2)	1.56 (0.90–2.72)	
High-risk unit attendance				
Yes	30 (50.8)	29 (49.2)	1	1
No	61 (31.3)	134 (68.7)	2.27 (1.26–4.11)*	2.07 (0.77–5.60)
Hospital admission during pregnancy				
Yes	7 (25.9)	20 (74.1)	1	
No	84 (37.0)	143 (63.0)	0.60 (0.24–1.47)	
Private health insurance				
Yes	11 (52.4)	10 (47.6)	1	
No	68 (34.0)	132 (66.0)	2.14 (0.86–5.28)	
Exclusively public health follow-up				
Yes	72 (53.3)	132 (64.7)	1.12 (0.59–2.13)	
No	19 (38.0)	31 (62.0)	1	
Exclusively private health follow-up				
Yes	10 (62.5)	6 (37.5)	0.31 (0.11–0.88)*	0.57 (0.13–2.50)
No	81 (34.0)	157 (66.0)	1	1
Difficulty in attending to appointments				
Yes	12 (20.7)	46 (79.3)	2.78 (1.37–5.66)*	0.71 (0.24–2.06)
No	66 (42.0)	91 (58.0)	1	
Difficulty in scheduling appointments				
Yes	22 (23.4)	72 (76.6)	2.91 (1.60–5.29) ^&^	2.87 (1.18–6.99)*
No	56 (47.1)	63 (52.9)	1	1
Virtual consultations				
Yes	32 (25.4)	94 (74.6)	2.82 (1.59–4.98) ^&^	3.08 (1.28–7.40)*
No	46 (48.9)	48 (51.1)	1	1

Abbreviations: OR, odds ratio; SD, standard deviation.

* *p* < 0.05; ^&^ *p* < 0.001; **Adjusted analysis for confounding factors (age, social class, and isolation during the COVID-19 pandemic); Hosmer-Lemeshow = 0.494.

In the pandemic context, more than half of postpartum women reported partial social isolation, and less than 5% of them reported complete social isolation ( Table 4 ). In total, 31 women were suspected or diagnosed with COVID-19, with confirmed etiology in 9 patients (the remainder had not been diagnosed through laboratory tests, but through clinical and/or epidemiological course), and 2 were hospitalized during pregnancy (data not shown). The COVID-19 pandemic and its association with the adequacy of antenatal care is displayed in table 4 . We found no association of any variable with adequacy of antenatal care, even in the adjusted model. The model was considered highly suitable by the Hosmer-Lemenshow test (0.991).

**Table 4:** COVID-19 pandemic and adequacy of antenatal care

Variables	Antenatal		Crude OR (CI95%)	Adjusted OR** (CI95%)
	Adequate n (%)	Inadequate n (%)		
Avoided crowds				
Yes	76 (36.9)	130 (63.1)	1	1
No	4 (19.0)	17 (81.0)	2.49 (0.81-7.66)	2.38 (0.45-12.67)
Avoided close contacts				
Yes	74 (37.2)	125 (62.8)	1	1
No	6 (21.4)	22 (78.6)	2.17 (0.84-5.60)	1.52 (0.44-5.26)
Public transportation				
Yes	17 (26.6)	47 (73.4)	1.74 (0.92-3.30)	1.68 (0.81-3.49)
No	63 (38.7)	100 (61.3)	1	1
Worked				
Yes	45 (37.5)	75 (62.5)	1	
No	32 (34.0)	62 (66.0)	1.16 (0.66-2.04)	
Work outside of home				
Yes	23 (30.7)	52 (69.3)	1.36 (0.75-2.45)	
No	57 (37.5)	95 (62.5)	1	
Avoided leaving home				
Yes	66 (35.9)	118 (64.1)	1	
No	14 (32.6)	29 (67.4)	1.16 (0.57-2.35)	
Behavior during the pandemic				
Total isolation	2 (20.0)	8 (80.0)	2.40 (0.45-12.83)	0.25 (0.02-3.64)
Partial isolation	63 (35.6)	114 (64.4)	1.09 (0.53-2.21)	0.88 (0.39-1.98)
No isolation	15 (37.5)	25 (62.5)	1	1
Had COVID-19				
Yes	8 (25.8)	23 (74.2)	1.69 ((0.72-3.98)	1.30 (0.53-3.22)
No	73 (37.1)	124 (62.9)	1	1

Abbreviation: OR, odds ratio.

**Adjusted analysis for confounding factors (age, social class, and paid work); Hosmer-Lemeshow = 0.991.

## Discussion

In our sample, 35.8% of pregnant women received adequate antenatal care during the COVID-19 pandemic. According to official 2019 data, the percentage of women who had adequate or more than adequate antenatal care was 77.5% in Florianopolis, 79.3% in the South region, and 70.7% in overall Brazil. 16 The Southern region of Brazil has a significantly higher chance of offering adequate antenatal care. 12 We found a percentage of inadequate antenatal care of 17.4%, higher than the 14.8% official data for 2019 in Florianópolis. 16 Notwithstanding, the 2019 data were based on first antenatal care appointment up to 12 weeks as a criterion for adequacy, rather than up to 16 weeks, as in our study. Even applying a less rigid standard, our findings suggest that fewer women had adequate or more than adequate antenatal care during the COVID-19 pandemic in Florianopolis when compared with the previous year in the same state, and in the overall country. Thus, we concluded that a decrease in the adequacy of antenatal care occurred in Florianopolis during the 2020 COVID-19 pandemic.

Skin color, nationality and primiparity were significant for antenatal care adequacy, with only skin color remaining in the adjusted analysis. Women with black or brown skin were 2.99 times more likely to have inadequate antenatal care, as already described in the literature. 2 3 4 Non-white skin color is a known risk for delayed first antenatal care appointment as well as lower rates of complementary exams during pregnancy follow-up. 3 This is especially relevant, considering that black women have the highest maternal mortality within the obstetric population in Brazil, along with more barriers to health access during the COVID-19 pandemic. 17

Foreign women had less access than adequate antenatal care and a higher chance of inadequate care in our sample, though the association was not maintained in the logistic regression. Previously, pregnant Haitians living in Brazil had fewer antenatal consultations when compared to Brazilian women, due to language barriers, prejudice or intolerance, and irregular/illegal documentation. 18 However, studies on the topic are scarce.

Primiparous women had a lower proportion of inadequate antenatal care. Multiparous women may be less assiduous to antenatal consultations because they have been pregnant before. Additionally, barriers to health access, adversity of maternal social context, or previous negative experiences with the health system may play a role favoring primiparity over multiparity on antenatal care adherence. 2 We also hypothesized that the closing of schools and day-care centers during the pandemic posed further difficulties for pregnant women with older children.

Several aspects that have been previously associated with the adequacy of antenatal care were not observed in our sample, that is, maternal age, literacy, income, and comorbidities. Previous findings showed higher antenatal adequacy associated with older maternal age, 6 19 and in women with longer formal education. 13 19 Low income and unemployment are risk factors for antenatal care inadequacy, before and during the pandemic. 13 20 Women with comorbidities or previous in-hospital treatment appear to have higher antenatal care adequacy rates. 4

We found 13.8% of reported alcohol consumption during pregnancy among participants, 11% of cigarette consumption, and 6% of drug use, higher rates than previously reported data from Brazil. 21 The COVID-19 pandemic may have intensified psychoactive substances utilization, such as alcohol, tobacco, and others. 22 The consumption of substances was not associated with adequacy of antenatal care.

In our sample, 63% of women began antenatal follow-up before 16 weeks, and 48.4% before 12 weeks. In comparison, before the pandemic, 75.8% and 50% of women had early antenatal care attendance at 16 and 12 weeks of pregnancy, respectively. 12 14 In our sample, 34.3% of women had at least 6 appointments, lower numbers than the 75% reported in previous years. 6 12 Early beginning of antenatal follow-up was delayed, but the number of appointments suffered a more severe decrease, maybe due to health system organization during the pandemic in Brazil.

After evidence of coronavirus community transmission in the country, the Brazilian Ministry of Health released recommended (not mandatory) COVID-19 contention measures, subsequently locally regimented as quarantine regimens. 23 Officially, women's health care should not be discontinued. However, non-emergency appointments have been postponed or cancelled in Florianópolis. Regarding antenatal care, high-risk pregnancy follow-up, first consultations, and follow-up after the 36 ^th^ week of pregnancy were maintained, and the remainder offered through virtual appointments. 24

Approximately 72% of participants were routinely tested for HIV, syphilis, and urine at the third trimester of pregnancy. Nationally, the percentage varies from 21.6 to 25.4%. 12 25 In our sample, one third or more of the women reported extra expenses during pregnancy, a common fact in Brazilian obstetric care. Previously, many pregnant women claimed to have paid for appointments and complementary tests in Fortaleza, 26 and for obstetric ultrasound in São Paulo. 27 Such unexpected expenditures suggest lower access to health care.

Women who attended high-risk antenatal units had higher antenatal care adequacy. For 15 participants referred to high-risk units without receiving the care (reasons obscured due to study design), the association with better adequacy was not observed. Before the pandemic, the percentage of high-risk pregnancies in Brazil was 15%, and many pregnant women reported delays in accessing their referral units. 26 Amidst the COVID-19 pandemic, the number of antenatal care appointments may have been reduced for women with comorbidities also. 19

We estimated access to antenatal care during the COVID-19 pandemic by identifying obstacles on receiving or scheduling appointments, as well having virtual consultations. One quarter of postpartum women reported difficulties in reaching scheduled consultations, which did not remain after the adjusted analysis. Approximately half of the women (44%) reported troubles in scheduling appointments, increasing the risk of inadequate antenatal care by 3 times. Three participants declared that the difficulty in scheduling was the reason for abandoning antenatal follow-up.

Even though virtual consultations were significantly associated with inadequate antenatal care, it is not possible to distinguish whether they were the cause or consequence of the association. Remote consultations are described as a viable socioeconomic alternative during the COVID-19 pandemic. 28 Thus, some guidelines state that they should be encouraged when physical examination is not necessary, and when pregnancy does not have risk factors. However, there are concerns about the lack of light technologies that depend on human contact, such as physical examination, blood pressure assessment, edema evaluation, and uterine fundal height measurement. 20 28 Virtual consultations may be a valid and necessary alternative and can assure access under special circumstances. Evidently, when health access is impaired, it is more appropriate to have virtual appointments than to have none. However, there is a need to develop validated protocols and systems for assessing virtual consultations quality, as well as guidelines identifying situations of necessary face-to-face assistance.

Only a few women adopted social isolation in our sample. Although Brazil appears as the country with the highest number of maternal deaths related to COVID-19, 29 the government failed in adopting or encouraging social isolation measures. Horizontal social isolation was not encouraged in Brazil. Isolation of contacts was also compromised due to limited tests availability at the time of data collection, corroborated by the small number of women tested to confirm COVID-19 diagnosis, as well as low adherence to social isolation in our sample. Our finding of few hospital admissions could be explained due to the fact that the chosen facility was not a local reference for COVID-19 cases. Access to primary care in Brazil faces adversity since long before the incoming pandemic. 30 Once antenatal care adequacy was not associated with social isolation, COVID-19 contamination, or difficulties in scheduling appointments, barriers to access health care emerge as the bottom line. Some authors have suggested COVID-19 suspicion or diagnosis might be associated with antenatal care adequacy, 7 8 which was not evidenced in our sample. Our study has limitations. First, it is difficult to compare studies on the subject, due to lack of criteria to analyze antenatal care adequacy, and the wide differences within the findings. 1 12 13 31 Usually, authors assess adequacy through quantitative and restricted criteria, and quality of care is not evaluated. 3 12 Secondly, it was not possible to determine sample selection bias, which could have significantly altered the results. However, according to the sample size calculation, the number of completed questionnaires was more than double needed to assess the outcome, and the results were in agreement with the published data. 1 4 12 Our findings suggest the need for further studies on the subject for better evaluation and qualification of obstetric care in Brazil.

Our study showed a decrease in the number of women who had access to adequate antenatal care during the COVID-19 pandemic in Florianopolis, a location with broad coverage of a primary care access initiative in Brazil (the Family Health Program). 32 It is estimated that little as 10% decrease in health care access for pregnant women during the COVID-19 pandemic in low-income countries may cause a considerable increase in obstetric complications and maternal deaths, affecting more than one million people worldwide. 19

Universal and qualitative health care is achievable by providing continuous professional training, compliance with technical standards, and adequate management of available health resources, prioritizing vulnerable women. 13 Access to qualified antenatal care should be favored, despite challenges of lack of resources and the pandemic pressure over the already weakened Brazilian health system. 20

## Conclusion

In a sample of pregnant women from a teaching hospital in Florianópolis during the COVID-19 pandemic, antenatal care was considered adequate for 35.8%, intermediate for 46.8%, and inadequate for 17.4% of the public. There were significant differences on adequacy of antenatal care regarding skin color: white and Asian women had a higher proportion of adequate antenatal care, while black and brown-skinned women had higher proportions of intermediate antenatal care ( *p* = 0.001). As for parity, the majority of both primiparous and multiparous women had intermediate antenatal care, though a higher number of multiparous women had inadequate care ( *p* = 0.037). Women with foreign nationality had inadequate antenatal care more frequently ( *p* = 0.044). The following variables were significantly associated with inadequate antenatal care: black or brown skin color (odds ratio [OR] 2.99 (95% confidence interval [CI] 1.49–6.00), difficulty in scheduling appointments (OR 2, 87 (CI 1.18–6.99), and virtual consultations (OR 3.08- CI 1.28–7.40). The increase of at least 1 appointment in the total number of antenatal consultations increased the chance of adequate antenatal care by 34% (OR 0.66; 95% CI: 0.55–0.79), and each delay in one week before the beginning of antenatal follow-up increased the chance of inadequate prenatal care by 1.17 times (95% CI: 1.06–1.29). Only 4.4% of women consistently practiced social isolation during pregnancy, and 31 women were diagnosed with COVID-19 during pregnancy. None of those variables were associated with the adequacy of antenatal care.
